# Intestinal, liver and lipid disorders in genetically obese rats are more efficiently reduced by dietary milk thistle seeds than their oil

**DOI:** 10.1038/s41598-021-00397-1

**Published:** 2021-10-22

**Authors:** Paulina M. Opyd, Adam Jurgoński

**Affiliations:** 1 Department of Animal Nutrition and Feed Science, University of Warmia and Mazury in Olsztyn, Oczapowskiego 5 Str., 10-719 Olsztyn, Poland; 2 Institute of Animal Reproduction and Food Research, Polish Academy of Sciences, Tuwima 10 Str., 10-748 Olsztyn, Poland; 3 Department of Biological Function of Food, Institute of Animal Reproduction and Food Research, Polish Academy of Sciences, Tuwima 10 Str., 10-748 Olsztyn, Poland

**Keywords:** Experimental models of disease, Obesity

## Abstract

We hypothesized that milk thistle seed or seed oil dietary supplementation reduces intestinal, liver and lipid disorders specific to genetic obesity, and the seeds can be more efficient in doing so. Lean and obese male Zucker rats were allocated to 4 groups: the lean (LC) and obese control (OC) groups fed a standard diet and the other 2 obese groups fed a diet supplemented with milk thistle seed oil (O + MTO) or milk thistle seeds (O + MTS). After 5 weeks of feeding, the cecal SCFA pool was slightly and significantly lower in OC and O + MTO compared with LC and O + MTS. The liver fat content was greater in OC, O + MTO and O + MTS compared with LC; however, it was significantly lower in O + MTS than in OC and O + MTO. The plasma cholesterol was greater in OC compared with LC, O + MTO and O + MTS; however, it was significantly greater in O + MTO and O + MTS compared with LC. The plasma bilirubin was detected in OC and O + MTO, whereas it was not present in LC and O + MTS. Milk thistle seeds can improve fermentation events in the distal intestine and reduce other disorders specific to genetically obese rats, and the seed PUFAs are responsible for that to a lesser extent.

## Introduction

*Silybum marianum* L., commonly called milk thistle, is currently one of the most important species of medicinal plants grown on herbal plantations^1^. Various parts of the plant, including leaf, flower and fruits, are rich in nutritional and bioactive compounds^2,3^, but its seeds have been the most popular in recent time. Like many other seeds from herbal plants, milk thistle seeds contain bioactive compounds, mainly silymarin, which represents ~ 4% of the seeds dry weight and is an isomeric mixture of unique flavonoid complex—flavonolignans^4^. This complex includes isosilybin, silychristin, isosilychhristin, silydianin and silimonin; however the main bioactive component is silybin, which comprising approximately 50–70% of milk thistle seed extracts^5^. Despite their low bio-availability, flavonolignans extracted from milk thistle seeds have been proven as hepatoprotective agents, and also possess anti-oxidative, antifibrotic, anti-inflammatory, membrane stabilizing, immunomodulatory and chemopreventive properties^6,7^. However, milk thistle seeds are also rich in nutrients and dietary fiber, the content of which is 42%^8^. The seeds contain ~ 20% protein that is rich in most of essential amino acids, especially in leucine, valine and lysine, but with the exception of tryptophan, which is not present in the seeds at all^9,10^. Milk thistle seeds also contain significant amounts of oil (20–23%), which is considered as a by-product of silymarin industrial production^11^. This oil is rich in unsaturated fatty acid and can also be a valuable foodstuff^12^, because it is rich in linoleic acid and oleic acid, the proportions of which can be up to 54% and 24% of total fatty acid, respectively^13^. Furthermore, some authors have reported the presence of other bioactive compounds in the oil fraction of this seeds, such as phytosterols (*β*-sitosterol, *δ*-7-stigmasterol, stigmasterol, campesterol) and *α*-tocopherol^12,14^. Interestingly, although the available data suggests that only traces of flavonolignans are present in the milk thistle seed oil (ca. 500 μg/ml)^15^, its antioxidative, cytoprotective and hepatoprotective activities have been also reported in vitro and in vivo^12,16,17^.

Obese Zucker rats are one of the best known and most widely used animal models of genetic obesity. Recessive homozygotes of these animals (*fa/fa*) have mutation in the leptin receptor, which constitutes a molecular background of their obesity^18^. As a result of this mutation, obese Zucker rats have disturbed regulation of food intake, which is caused by the lack of functional leptin receptors in the central nervous system. Despite the high level of leptin in the blood, they are insensitive to this hormone, which in physiological conditions and in the lean Zucker phenotype (*Fa/?*) reaches the brain and restricts food intake, among others^19^. Thus, obese Zucker rats are characterized by disturbed energy homeostasis and drastic weight gain associated with disorders similar to those seen in human metabolic syndrome, such as fatty liver, dyslipidemia, insulin resistance, chronic inflammation, and some endocrine disorders^20^.

The consumption of herbal-based supplements, which are believed to have beneficial effects on human health is more and more popular around the world. Milk thistle-based supplements are an important part of this trend, which is mostly a result of research focused around the hepatoprotective activities of silymarin^6,7^. Studies on the nutritional properties and health-related effects of milk thistle seeds and their oil are relatively scarce. Thus, the aim of this study was to compare the effects of dietary supplementation with milk thistle seeds and milk thistle seed oil on the development of metabolic disorders in genetically obese Zucker rats. We hypothesized that milk thistle seed or milk thistle seed oil supplementation can attenuate genetically determined disorders of the gastrointestinal tract, liver and lipid metabolism and that the former is more effective due to a wider range of bioactive compounds present in the seeds.

## Materials and methods

### Preparation and chemical composition of milk thistle seed and milk thistle seed oil

Whole milk thistle seeds (*Silybum marianum* L.) were from the Intenson Europe Ltd. company (Karczew, Poland), whereas unrefined, cold-pressed milk thistle seed oil was from the Ol’Vita company (Panków, Poland). The chemical composition of milk thistle seeds and milk thistle seed oil was determined by an accredited research laboratory (Nuscana, Mrowino, Poland) based on the official procedures of AOAC^21^. In the seeds, the dry matter (DM) and ash content were determined by the gravimetric method after drying at 100 °C and 60 °C, respectively. Total dietary fiber was determined by the enzymatic–gravimetric method, crude protein was determined by the Kjeldahl method, crude fat was determined by the Soxhlet extraction method and then, the nitrogen-free extract was calculated. The fatty acid profile of the oil and the oil fraction extracted from the seeds was determined by gas chromatography with flame-ionization detection after previous conversion of the fatty acids into respective methyl esters. All studies of milk thistle seeds were carried out in accordance with the relevant institutional and international guidelines and regulations The basic chemical composition of the seeds and the fatty acid profile of the seeds and oil are shown in Table 1.

**Table 1:** Chemical composition of milk thistle seeds and seed oil.

	Milk thistle seed oil	Milk thistle seeds
Dry matter (DM), %	–	92.8 ± 0.10
Ash, % DM	–	4.20 ± 0.01
Dietary fibre, % DM	–	44.8 ± 1.11
Nitrogen-free extract, % DM	–	5.2 ± 0.02
Crude protein, % DM	–	13.9 ± 0.41
Crude fat, % DM	–	24.8 ± 0.33
**Fatty acid profile (%)**		
Palmitic acid (16:0)	6.98 ± 0.01	6.98 ± 0.01
Stearic acid (18:0)	4.44 ± 0.00	4.86 ± 0.01
Oleic acid (18:1 *n*-9)	22.84 ± 0.07	23.8 ± 0.00
Linoleic acid (18:2 *n*-6)	55.12 ± 0.07	53.0 ± 0.03
Arachidic acid (20:0)	2.55 ± 0.01	2.92 ± 0.01
Gondoic acid (20:1 *n*-9)	0.82 ± 0.00	0.96 ± 0.00
*α*-Linolenic acid (18:3 *n*-3)	0.13 ± 0.00	0.12 ± 0.00
Behenic acid (22:0)	1.87 ± 0.01	2.11 ± 0.01
Lignoceric acid (24:0)	0.50 ± 0.01	0.60 ± 0.01
**Calculated fatty acid content (%)**		
SFA	16.3 ± 0.01	17.5 ± 0.04
MUFA	23.7 ± 0.04	24.7 ± 0.00
**PUFA**	55.3 ± 0.04	53.1 ± 0.03
*n*-3	0.13 ± 0.00	0.12 ± 0.00
*n*-6	55.1 ± 0.03	53.0 ± 0.03

*DM* dry matter, *MUFA* monounsaturated fatty acid, *PUFA* polyunsaturated fatty acid, *SFA* saturated fatty acid.

Values are means ± SD (n = 3).

### Animals, diets and experimental design

The animal protocol employed in this experiment was in compliance with European guidelines for the care and use of laboratory animals and was approved by the Institutional Animal Care and Use Committee in Olsztyn, Poland (permission number: 37/2017). This research was done in compliance with the ARRIVE guidelines and regulations. The feeding experiment was conducted on 31 lean (*Fa/?*) and obese (*fa/fa*) 8-week-old male Zucker rats allocated to one lean and three obese groups. Initial body weight (BW) of rats is shown in Table 3. The rats were individually housed in plastic cages and a controlled environment (12-h light–dark cycle, a temperature of 21 ± 1°C, relative humidity of 55 ± 10% and 15 air changes per hour). For 35 days, each group was fed a modified version of the semipurified casein diet recommended for rodents by Reeves^22^. The lean control (LC) and obese control (OC) groups were fed a diet containing casein, cellulose and rapeseed (canola type) and palm oil (4% of each oil) as the source of protein, fiber and fat, respectively. The other two obese groups were fed a modification of the standard diet in which milk thistle seed oil was added at the expense of palm oil (4% diet; O + MTO group) or ground milk thistle seeds were added at the expense of palm oil, cellulose and casein (16.15% diet; O + MTS group). Milk thistle seeds were ground for 1 min at a temperature below 37°C prior to their inclusion in the diet. All diets had the same proportion of protein (18%), fat (8.3%) and fiber (8%). Diets fed to the O + MTO and O + MTS groups had also similar fatty acid profiles, including a more than two-fold increase in the proportion of PUFAs that originated either from milk thistle seeds or milk thistle seed oil. After preparation, the diets were stored at 4°C under limited access of oxygen. This experimental design allowed us to assess the extent to which the lipid fraction of milk thistle seeds contributes to the health effects of their consumption and was based on a previous study, in which male Zucker rats and oilseeds were used^23^. The detailed composition of the diets, which were freely available to rats for the entire experimental period, is shown in Table 2.

**Table 2:** Composition of the diets fed to rats (g/100 g diet).

Components	Group			
	LC	OC	O + MTO	O + MTS
Casein^a^	20.00	20.00	20.00	17.47
dl-methionine	0.3	0.3	0.3	0.3
Rapeseed oil^b^	4	4	4	4
Palm oil^c^	4	4	–	–
Milk thistle seed oil^d^	–	–	4	–
Milk thistle seed^d^	–	–	–	16.15
Corn starch	49.00	49.00	49.00	46.61
Sucrose	10	10	10	10
Cellulose	8.00	8.00	8.00	0.77
Mineral mix	3.5	3.5	3.5	3.5
Vitamin mix	1	1	1	1
Choline chloride	0.2	0.2	0.2	0.2
**Calculated content (%)**				
Protein	18.0	18.0	18.0	18.0
**Fat**	8.3	8.3	8.3	8.3
SFAs	2.11	2.11	1.71	1.75
MUFAs	4.09	4.09	2.99	3.03
PUFAs	1.40	1.40	2.91	2.83
*n*-3	0.33	0.33	0.17	0.17
*n*-6	1.08	1.08	2.74	2.66
Fibre	8.0	8.0	8.0	8.0

*MUFA* monounsaturated fatty acid, *PUFA* polyunsaturated fatty acid, *SFA* saturated fatty acid, *LC* lean control, *OC* obese control, *O + MTO* obese fed a diet containing milk thistle oil, *O + MTS* obese fed a diet containing milk thistle seeds.

^a^Casein preparation (g/100 g): crude protein, 88.70; crude fat, 0.3; ash, 2.0; water, 8.0.

^b^Canola type oil with the following fatty acid profile (%): SFAs, 6.49; MUFAs, 62.9 including oleic acid, 56.9; PUFAs, 25.9 including linoleic acid and *α*-linolenic acid, 17.5 and 8.06, respectively.

^c^Fatty acid profile (%): SFAs, 46.3 including palmitic acid, 40.8; MUFAs, 39.4 including oleic acid, 39.1; PUFAs, 9.21 including linoleic acid, 9.1.

^d^Chemical composition in Table 1.

### Analysis of body composition in rat

At the end of the experimental feeding, the body lean and fat masses of the rats were determined by time-domain NMR using the Minispec LF 90II analyzer (Bruker, Karlsruhe, Germany). The method relies on transmitting various radio frequency pulses into soft tissues to reorient the nuclear magnetic spins of the hydrogen and then detects radio frequency signals generated by the hydrogen spins from these tissues. The contrast in relaxation times of the hydrogen spins found between adipose tissue and water-rich tissues is used to estimate their masses within the body.

### Collection of biological material and analytical procedures

After 5 weeks of experimental feeding, rats were anesthetized with a mixture of xylazine and ketamine in physiological salt (10 mg and 100 mg/kg BW, respectively). Each animal was then weighed, and the abdomen was cut open. Blood was subsequently collected from the *vena cava* into heparinized tubes, centrifuged for 10 min at 380×*g* and 4°C, and the obtained plasma was then frozen until analysis. Next, the small intestine, cecum, colon, and liver were removed, weighed, and frozen using liquid nitrogen or were used for further treatment.

Samples of fresh ileal, cecal, and colonic digesta were collected, and the pH values were measured using a microelectrode and pH/ION meter (model 301, Hanna Instruments). The ammonia concentration in the fresh caecal digesta was extracted, trapped in a solution of boric acid and then quantified by direct titration with sulphuric acid in Conway dishes according to the method described by Hofirek and Haas^24^. The SCFA concentration was determined in cecal digesta after storage at − 20°C using GC (Shimadzu Co.) and a capillary column (SGE BP21, 30 m × 0.53 mm; SGE Europe Ltd.) as previously described^25^.

Liver lipids were extracted according to the method of Folch et al.^26^ with previously described modifications^27^. Briefly, the liver slice was homogenized with a 2:1 mixture of chloroform–methanol (0.2 g in 4 mL of mixture) using a high-performance homogenizer (IKA T25 digital ULTRA-TURRAX^®^,Wilmington, NC, USA) and then centrifuged at 15,000×*g* for 10 min. The supernatant was washed with 0.8 mL of distilled water, vortexed and centrifuged for 15 min (2500 × *g*). After removing the upper phase, the lower phase containing lipids was evaporated under a nitrogen stream at 37°C. Lipids obtained in this way were then dissolved with 2.88 mL of chloroform, and liver cholesterol and triglyceride concentrations were determined spectrophotometrically in the extracted lipid phase using reagents from Alpha Diagnostics Ltd.

Malondialdehyde was determined spectrophotometrically (at 532 nm) in the liver based on a procedure developed by Botsoglou et al.^28^ and its content was expressed in μg malondialdehyde per g of liver.

The plasma concentration of cholesterol (total and its HDL and non-HDL fractions), triglycerides, total and direct bilirubin, albumin and the plasma activities of aspartate transaminase (AST), alanine transaminase (ALT), and alkaline phosphatase (ALP) were determined using an automatic biochemical analyzer (Pentra C200, Horiba Ltd.). Indirect bilirubin was calculated as the difference between total and direct bilirubin.

### Gene expression analysis in the liver

The expression of genes associated with the lipid metabolism (PPARα, PPARγ and SREBP1c) was analyzed on the mRNA level according to the previously described method^27^. Briefly, RNA was extracted from the liver using TRI Reagent solution according to the manufacturer’s instructions (Thermo Fisher Scientific, Waltham, MA, USA). Quantity and quality of RNA were measured spectrophotometrically using a NanoDrop1000 (Thermo Fisher Scientific) and agarose gel electrophoresis, respectively. cDNA was synthesized from 500 ng of total RNA using a High-Capacity cDNA Reverse Transcription Kit with RNase Inhibitor (Applied Biosystem, Waltham, MA, USA). *β*-actin was selected as a reference gene. The mRNA expression levels of peroxisome proliferator-activated receptor *α* (PPAR*α*), peroxisome proliferator-activated receptor *γ* (PPAR*γ*), sterol regulatory element binding protein 1c (SREBP1c), and *β*-actin were analyzed using Single Tube TaqManVR Gene Expression Assays (Life Technologies, CA, USA). Amplification was performed using a 7900HT Fast Real-Time PCR System under the following conditions: initial denaturation for 10 min at 95°C; 40 cycles of 15 s at 95°C and 1 min at 60°C. Each run included a standard curve based on aliquots of pooled liver RNA. All samples were analyzed in duplicates. The mRNA expression levels of PPAR*α*, PPAR*γ*, and SREBP1c were normalized to *β*-actin and multiplied by 10.

### Statistical analysis

The results are expressed as the mean ± standard error of the mean (SEM), except for the chemical composition of milk thistle seeds and milk thistle seed oil, the results of which were is expressed as the mean ± standard deviation (SD). One-factor analysis of variance (ANOVA) and Duncan’s multiple range post hoc test were used to determine significant differences between groups (*P* ≤ 0.05). If the ANOVA assumptions were not met, the Kruskal–Wallis 1-factor ANOVA by ranks was used followed by Dunn’s post hoc test with the Bonferroni correction (*P* ≤ 0.05). All calculations were performed using STATISTICA version 12 (StatSoft Corp.).

## Results

The chemical composition of milk thistle seeds and milk thistle seed oil is shown in Table 1. The main component of the seeds was dietary fiber which constituted 44.8% DM. Milk thistle seeds contained also a considerable amounts of fat and protein which were 24.8% and 13.9% DM, respectively. The fatty acid proportion was similar between the seeds and oil. Both milk thistle seeds and milk thistle seed oil were rich in polyunsaturated fatty acids (PUFAs; 53.1% and 55.3% of total fatty acids, respectively), including 53.0% and 55.1% of linoleic acid as the main component and some trace amounts of α-linolenic acid (0.12% and 0.13%, respectively). Both seeds and oil also contained similar proportion of monounsaturated fatty acids (MUFAs; 24.7% and 23.7% of total fatty acids, respectively), including 23.8% and 22.8% of oleic acid, respectively. The overall proportion of saturated fatty acids (SFAs) in milk thistle seeds and milk thistle seed oil was 17.5% and 16.3% of total fatty acids, respectively, including palmitic acid as the main SFA both in the seeds and oil (6.98% each).

The diet intake, body weight and body composition of lean and obese Zucker rats fed a diet supplemented with milk thistle seeds and milk thistle seed oil are shown in Table 3. The initial BW was to a similar degree higher in all obese groups compared with the LC group (P ≤ 0.05). The diet and calorie intake calculated on a daily basis was also similarly higher in the obese groups compared with the LC group (P ≤ 0.05). As a result, rats from all obese groups gained more body weight and body fat percentage, whereas the body lean percentage was lower compared with the LC group (P ≤ 0.05).

**Table 3:** Diet intake, body weight and body composition of lean and obese Zucker rats fed a diet containing milk thistle seed oil (+ MTO) or milk thistle seeds (+ MTS) for 5 weeks.

Index	Group				ANOVA *P* value
	LC	OC	O + MTO	O + MTS	
Initial BW, g	168 ± 1.48^b^	210 ± 6.31^a^	206 ± 6.64^a^	210 ± 3.43^a^	< 0.001
Diet intake, g/day	16.0 ± 0.455^b^	24.0 ± 0.960^a^	23.3 ± 0.51^a^	24.09 ± 0.51^a^	< 0.001
Calorie intake, kcal/day	57.2 ± 1.63^b^	86.0 ± 3.44^a^	83.4 ± 1.82^a^	84.3 ± 1.79^a^	< 0.001
Final BW, g	291 ± 6.57^b^	411 ± 12.3^a^	404 ± 5.89^a^	409 ± 8.00^a^	< 0.001
BW gain, g	123 ± 6.74^b^	201 ± 8.58^a^	199 ± 6.15^a^	198 ± 6.53^a^	< 0.001
Body fat, %	20.9 ± 1.04^b^	54.3 ± 0.513^a^	52.3 ± 0.480^a^	52.8 ± 0.749^a^	< 0.001
Body lean, %	62.4 ± 0.736^a^	38.7 ± 0.247^b^	39.6 ± 0.360^b^	39.3 ± 0.585^b^	< 0.001

Values are means ± SEMs, *n* = 7–8. Labeled means in a row without a common letter (a,b) differ at *P* ≤ 0.05 (Duncan’s or Dunn’s post hoc test).

*BW* body weight, *LC* lean control, *OC* obese control, *O + MTO* obese fed a diet containing milk thistle seed oil, *O + MTS* obese fed a diet containing milk thistle seeds.

The effects of milk thistle seeds and milk thistle seed oil on markers of the gastrointestinal tract function in rats are shown in Table 4. In the O + MTO group, the mass of the small intestine relative to BW was significantly lower compared with the LC group (P ≤ 0.05), whereas the pH of ileal digesta was comparable among all groups (P > 0.05). Neither the rat phenotype nor the tested experimental factors had an influence on the cecal digesta mass nor ammonia concentration in the cecal digesta (P > 0.05). The mass of cecal and colonic empty segments was lower (P ≤ 0.05), whereas the colonic digesta mass was higher (P ≤ 0.05) in all obese groups compared with the LC group. The pH value of the cecal digesta was higher after dietary supplementation with the milk thistle seed oil (O + MTO group, P ≤ 0.05) compared with the LC and OC group, whereas the digesta pH in the O + MTS group was lower compared to the O + MTO and OC group (P ≤ 0.05). The colonic digesta pH was also higher in the obese control group compared with the LC group (P ≤ 0.05), and also in this case its value was lowered in the milk thistle seed-supplemented group (O + MTS group) being comparable with that of the LC group. The total SCFA concentration calculated on a gram of cecal digesta did not differ among all groups (P = 0.072), but the SCFA pool calculated as their concentration in the total digesta mass was affected by experimental factors (P < 0.005). The SCFA pool was significantly lower in the O + MTO group compared with the LC group (P ≤ 0.05), whereas in the milk thistle seed-supplemented group the pool was comparable with that of the LC and OC group (P < 0.05). The concentrations of individual SCFA in the cecal digesta were influenced both by dietary milk thistle seeds and milk thistle seed oil. The acetate concentration was lower in the O + MTO group compared to both control groups (LC and OC group; P ≤ 0.05), whereas the propionate concentration was lower compared with the LC group (P ≤ 0.05). The propionate concentration in turn, like the isobutyrate and isovalerate concentration, was higher in the O + MTS group compared to all other groups (P ≤ 0.05). The valerate concentration was lower in all obese groups compared with the LC group (P ≤ 0.05). As a result the aforementioned differences, the SCFA concentration of putrefactive origin calculated as the sum of isobutyrate, isovalerate and valerate was lower in the O + MTS group compared with the LC and OC group (P ≤ 0.05).

**Table 4:** Markers of the gastrointestinal tract function in lean and obese Zucker rats fed a diet containing milk thistle seed oil (+ MTO) or milk thistle seeds (+ MTS) for 5 weeks.

Index	Group				ANOVA *P* value
	LC	OC	O + MTO	O + MTS	
**Small intestine**					
Mass with digesta, g/100 g BW	2.03 ± 0.022^a^	1.93 ± 0.069^a,b^	1.85 ± 0.020^b^	1.92 ± 0.043^a,b^	< 0.05
pH of digesta	6.98 ± 0.108	7.13 ± 0.120	7.18 ± 0.150	7.07 ± 0.110	NS
**Cecum**					
Mass of empty segment, g/100 g BW	0.162 ± 0.019^a^	0.109 ± 0.004^b^	0.090 ± 0.000^b^	0.100 ± 0.003^b^	< 0.001
Digesta mass, g/g cecum	3.71 ± 0.365	3.00 ± 0.237	3.12 ± 0.170	3.40 ± 0.205	NS
pH of digesta	7.40 ± 0.047^b,c^	7.45 ± 0.050^b^	7.64 ± 0.050^a^	7.31 ± 0.025^c^	< 0.001
Ammonia, mg/g digesta	0.214 ± 0.01	0.235 ± 0.012	0.210 ± 0.010	0.210 ± 0.009	NS
**SCFA concentration, µmol/g digesta**					
Acetate	39.9 ± 2.40^a^	38.9 ± 1.80^a^	32.5 ± 1.48^b^	37.7 ± 1.82^a,b^	< 0.05
Propionate	11.8 ± 0.764^b^	9.92 ± 0.976^bc^	9.13 ± 0.690^c^	14.5 ± 0.835^a^	< 0.001
Isobutyrate	1.17 ± 0.061^a^	1.06 ± 0.035^a^	1.02 ± 0.090^a^	0.750 ± 0.047^b^	< 0.001
Butyrate	6.45 ± 0.683	4.94 ± 0.329	5.02 ± 0.580	4.58 ± 0.544	NS
Isovalerate	1.09 ± 0.052^a^	1.10 ± 0.057^a^	1.06 ± 0.090^a^	0.680 ± 0.031^b^	< 0.001
Valerate	1.26 ± 0.064^a^	1.01 ± 0.078^b^	0.920 ± 0.090^b^	1.04 ± 0.055^b^	< 0.01
PSCFA^A^	3.36 ± 0.244^a^	3.18 ± 0.13^a^	3.00 ± 0.230^a,b^	2.47 ± 0.121^b^	< 0.01
Total	60.8 ± 3.79	57.0 ± 2.79	49.7 ± 1.95	58.7 ± 3.24	NS
**SCFA profile, %**					
Acetate	65.9 ± 0.859^b^	68.4 ± 0.764^a^	65.4 ± 0.720^b^	64.5 ± 0.945^b^	< 0.05
Propionate	19.8 ± 1.31^b^	17.2 ± 1.02^b^	18.5 ± 1.32^b^	24.8 ± 0.479^a^	< 0.001
Butyrate	8.81 ± 1.45	8.74 ± 0.625	10.2 ± 1.18	6.53 ± 1.12	NS
PSCFA	3.36 ± 0.244^a^	3.18 ± 0.13^a^	3.00 ± 0.230^a,b^	2.47 ± 0.121^b^	< 0.01
SCFA Pool, µmol/total digesta mass	101 ± 11.2^a^	75.1 ± 5.52^a,b^	56.2 ± 2.75^b^	80.6 ± 4.89^a^	< 0.005
**Colon**					
Mass of empty segment, g/100 g BW	0.307 ± 0.013^a^	0.227 ± 0.014^b^	0.210 ± 0.010^b^	0.220 ± 0.009^b^	< 0.001
Digesta mass, g/g colon	1.20 ± 0.113^b^	1.72 ± 0.146^a^	1.70 ± 0.140^a^	1.94 ± 0.077^a^	< 0.05
pH of digesta	7.17 ± 0.07^b^	7.41 ± 0.077^a^	7.40 ± 0.030^a^	7.05 ± 0.044^b^	< 0.001

Values are means ± SEMs, *n* = 7–8. Labeled means in a row without a common letter (a,b,c) differ at *P* ≤ 0.05 (Duncan’s or Dunn’s post hoc test).

*BW* body weight, *NS* nonsignificant, *P* > 0.05, *PSCFA* SCFA of putrefactive origin, *SCFA* short-chain fatty acid, *LC* lean control, *OC* obese control, *O + MTO* obese fed a diet containing milk thistle seed oil, *O + MTS* obese fed a diet containing milk thistle seeds.

^A^The sum of isobutyrate, isovalerate and valerate.

**Figure 1:**
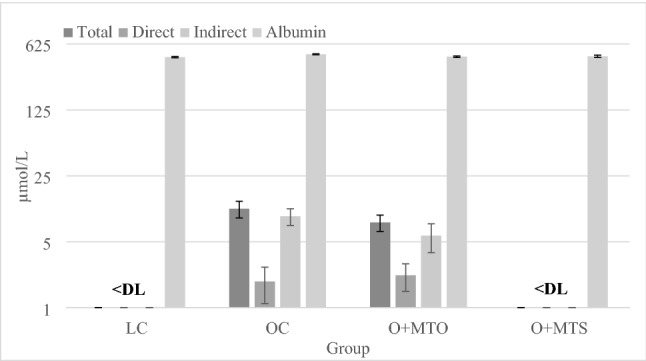
Plasma bilirubin and albumin concentration in lean and obese Zucker rats fed a diet containing milk thistle seed oil (+ MTO) or milk thistle seeds (+ MTS) for 5 weeks. Values are means ± SEMs, n = 7–8. *DL* detection limit for total bilirubin = 2.9 µmol/L and for direct bilirubin = 1.6 µmol/L, *LC* lean control, *OC* obese control, *O + MTO* obese fed a diet containing milk thistle seed oil, *O + MTS* obese fed a diet containing milk thistle seeds. Indirect bilirubin was calculated as the difference between total and direct bilirubin.

Markers of the liver function and lipid metabolism are shown in Table 5. The relative liver mass and the liver cholesterol and triglyceride contents were considerably higher in all obese groups compared to the LC group (P ≤ 0.05). The liver fat percentage was more than 2 times higher in all obese groups compared to the LC group; however, it was significantly lower in the O + MTS group compared to the other obese groups (P ≤ 0.05). The liver malondialdehyde content was higher in all obese groups compared to the LC group (P < 0.05). The hepatic expression of PPARγ and SREBP1c was lower in all obese groups compared with the LC group (P ≤ 0.05). The blood plasma AST, ALT and ALP activity was higher in all 3 obese groups compared with the LC group (P ≤ 0.05). The blood plasma concentration of total cholesterol and their HDL and non-HDL fractions was approximately 2 times higher in the OC group compared with the LC group (P ≤ 0.05); however, it was significantly lower in both dietary seed- and oil-supplemented groups (P ≤ 0.05), except the non-HDL cholesterol concentration in the O + MTO group, which was comparable with that of the OC and O + MTS group (P > 0.05). The plasma triglyceride concentrations were not affected by experimental factors (P > 0.05). The plasma albumin concentration was comparable among all groups (P > 0.05) (Fig. 1). The plasma concentration of total, direct and indirect bilirubin was similar between the OC and O + MTO group (P > 0.05), whereas it was not detectable in any form in the LC and O + MTS group (Fig. 1).

**Table 5:** Markers of the liver function and lipid metabolism in lean and obese Zucker rats fed a diet containing milk thistle seed oil (+ MTO) or milk thistle seeds (+ MTS) for 5 weeks.

Marker	Group				ANOVA *P* value
	LC	OC	O + MTO	O + MTS	
**Liver**					
Mass, g/100 BW	4.60 ± 0.097^b^	6.39 ± 0.240^a^	6.19 ± 0.250^a^	5.81 ± 0.315^a^	< 0.001
Fat, %	8.42 ± 0.496^c^	29.7 ± 1.22^a^	25.5 ± 2.44^a^	20.5 ± 1.71^b^	< 0.001
Triglycerides, mg/g	3.45 ± 0.319^b^	25.2 ± 1.82^a^	23.3 ± 2.13^a^	21.3 ± 2.00^a^	< 0.001
Cholesterol, mg/g	1.28 ± 0.084^b^	2.07 ± 0.075^a^	2.00 ± 0.100^a^	1.73 ± 0.186^a^	< 0.001
Malondialdehyde, µg/g	496 ± 13.2^b^	584 ± 26.3^a^	599 ± 26.7^a^	579 ± 19.8^a^	< 0.05
**mRNA expression**^A^					
PPAR*α*	7.44 ± 0.292	7.05 ± 0.691	6.91 ± 0.570	6.73 ± 0.898	NS
PPAR*γ*	13.5 ± 0.843^a^	9.67 ± 0.641^b^	8.74 ± 0.440^b^	8.75 ± 0.460^b^	< 0.001
SREBP1c	16.3 ± 0.979^a^	7.47 ± 0.831^b^	6.93 ± 0.650^b^	6.64 ± 1.10^b^	< 0.001
**Plasma**					
**Enzyme activity, U/L**					
AST	81.5 ± 4.94^b^	159 ± 14.2^a^	159 ± 20.4^a^	168 ± 14.2^a^	< 0.005
ALT	39.0 ± 1.86^b^	119 ± 8.63^a^	147 ± 20.0^a^	126 ± 12.8^a^	0.001
ALP	224 ± 5.89^b^	552 ± 62.7^a^	476 ± 68.0^a^	480 ± 78.7^a^	< 0.005
**Lipid profile, mmol/L**					
Total cholesterol	3.03 ± 0.085^c^	5.98 ± 0.142^a^	5.42 ± 0.190^b^	5.05 ± 0.206^b^	< 0.001
HDL cholesterol	0.956 ± 0.04^c^	1.74 ± 0.048^a^	1.35 ± 0.060^b^	1.33 ± 0.055^b^	< 0.001
Non-HDL cholesterol^B^	2.08 ± 0.054^c^	4.24 ± 0.123^a^	4.07 ± 0.130^a,b^	3.72 ± 0.166^b^	< 0.001
Triglycerides	3.57 ± 0.441	3.43 ± 0.442	2.96 ± 0.480	3.28 ± 0.422	NS

Values are means ± SEMs, *n* = 7–8. Labeled means in a row without a common letter (a,b,c) differ at *P* ≤ 0.05 (Duncan’s or Dunn’s post hoc test).

*AST* aspartate transaminase, *ALT* alanine transaminase, *ALP* alkaline phosphatase, *BW* body weight, *NS* nonsignificant, *P* > 0.05, *PPARα* peroxisome proliferator-activated receptor *α*, *PPARγ* peroxisome proliferator-activated receptor *γ*, *SREBP1c* sterol regulatory element binding protein 1c, *LC* lean control, *OC* obese control, *O + MTO* obese fed a diet containing milk thistle seed oil, *O + MTS* obese fed a diet containing milk thistle seeds.

^A^Gene expression/*β*-actin × 10.

^B^Non-HDL cholesterol was calculated as the difference between total cholesterol and HDL cholesterol.

## Discussion

The aim of this study was to compare milk thistle seed and milk thistle seed oil dietary supplementation on the development of metabolic disorders in obese Zucker rats. To assess the extent to which the lipid fraction could contribute to the health effects of milk thistle seed supplementation, it was necessary to precisely design semipurified diets. Therefore, detailed analysis of the chemical composition of milk thistle seeds and milk thistle seed oil was necessary (results in Table 1). The chemical composition of milk thistle seeds very much depends on geographical and environmental conditions^11^. The milk thistle seeds used in our study contained 44.8% DM of dietary fiber and this quantity was slightly higher than that of the flour made from milk thistle seeds by Bortlikova et al.^8^, which was 42.1%. The milk thistle seeds was moderately rich in protein (13.9% DM), especially when compared with the literature data on the protein content of milk thistle seeds that ranged from 20.1% to even 30.0% DM^8,11,29^. The fat content in the seeds was comparable with that reported by Aziz et al.^11^ (20–23%), and was also considerably lower than that reported by Bortlikova et al.^8^ (32.9%). The total proportion of PUFAs determined in our study in the milk thistle seeds and milk thistle seed oil (53.1% and 55.3% of total fatty acids, respectively) was higher than that of the milk thistle seed oil by Rokosik et al.^30^ and Zhang et al.^31^ (48.3% and 47.3% of total fatty acids, respectively). From the study design perspective, a very important question was that the fatty acid profile of the milk thistle seeds and milk thistle seed oil was similar. The main PUFA of the seeds and oil was linoleic acid (53.0% and 55.1%, respectively), and these results were generally in agreement with the literature^13,32^. Moreover, the seeds and oil were a relatively poor source of *α*-linolenic acid (0.12% and 0.13%, respectively) and similar proportions of this fatty acid were determined by Zarrouk et al.^32^ and Rokosik et al.^30^. Interestingly, Zhang et al.^31^ also reported that the milk thistle seed oil can be a good source of MUFAs, which can comprise over 30% of total fatty acids, and this proportion was higher than that of the present study both for the seeds and oil (24.7% and 23.7%, respectively). According to the literature, the main MUFA in the milk thistle seed oil is oleic acid^13^, which equaled 24.1% of total fatty acids, and comparable proportions of this acid was also determined in this study (23–24%).

Overconsumption of food is the main factor for obesity development. A good example of how quickly food overconsumption can lead to obesity are Zucker rats, in which hyperphagia cause visible obesity already in the first month of their life^20^. In the present study, obese Zucker rats were eating 1.5 times much diet per day than their lean counterparts, which at the end of the study, when they were 13 weeks old in total, make them more than 100 g heavier than their lean counterparts (Table 3). Moreover, the body fat percentage was more than 2 times higher in the obese rats than the lean rats, which partly took place at the expense of body lean percentage. Neither the seed nor oil supplementation were able to inhibit the development of obesity.

Obesity is directly associated with the development of gastrointestinal disorders within the body, especially with those related to the distal intestine, including quantitative and qualitative modifications in gut microbiota, irritable bowel syndrome, inflammatory bowel disease or colorectal cancer^33^. A previous study on Zucker rats showed that obese animals were characterized by lower bacterial counts and reduced microbiota metabolic activity in the hindgut than the lean phenotype^22,34^. In this study, a lower cecal SCFA pool was especially visible in the O + MTO group (Table 4), which indicates that the oil supplementation had some additional negative effects on the cecal microbiota. SCFAs, mainly acetate, propionate and butyrate, are the major end products of bacterial fermentation in the distal intestine and are considered intermediate nutrients for the body^35^. Fleming et al.^36^ showed that an elevated concentration of SCFA in the cecal digesta was associated with reduced pH of the digesta. This results are partly in agreement with our study, where, besides the reduced cecal SCFA pool, we observed increased colonic digesta pH in obese rats. To the best of our knowledge, this is the first study examining the effect of milk thistle seeds and milk thistle seed oil supplementation on hindgut fermentation. Interestingly, only dietary milk thistle seed supplementation increased the cecal SCFA pool, which was associated with a decrease of the digesta pH and could be considered as one of the main findings of this study. Those changes were most probably due to the cecal propionate, the concentration of which in the digesta of the O + MTO group was the highest among all tested groups. It is especially important, because propionate after its absorption into the bloodstream reaches the liver and can decrease hepatic lipogenesis^37^. Interestingly, dietary milk thistle seeds decreased isobutyrate and isovalerate concentration (Table 4), whereas the reduce of branched SCFA production is an indicator of reduced putrefaction in the distal intestine, because those types of fatty acids are important metabolites of this process^38^.

An elevated level of total bilirubin in blood plasma is usually associated with liver disorders. In the present study, obese Zucker rats (groups OC and O + MTO) have significant amounts of plasma total, direct and especially indirect bilirubin, whereas the plasma albumin concentration, which is the carrier for indirect bilirubin, was similar among all groups (Fig. 1). This undesirable increase of plasma bilirubin was accompanied by a several times higher plasma aminotransferase activities (ALT, AST) and ALP activity (Table 5), indicating on liver damage which explains the occurrence of hyperbilirubinemia. An increased level of indirect bilirubin is caused by compromised liver function and as a result of which hepatocytes cannot conjugate bilirubin with glucuronic acid^39^. Interestingly, Pizarro et al.^40^ reported that obese Zucker rats are characterized by decreased bile flow to the intestine. An increase of direct bilirubin caused by the disrupt of bile flow can regurgitate it back into the blood^39^. On the other hand, the intestinal metabolism of bilirubin includes its transformation into urobilinogen by intestinal bacteria^41^. As indicated in this and our previous study, obese Zucker rats are characterized by considerably reduced microbiota metabolic activity^22^, which may disturb the bilirubin conversion. Therefore, the bilirubin accumulation in the intestine may cause its increased reflux into the blood plasma^42^. Of note is that in this study dietary supplementation with milk thistle seeds prevented hyperbilirubinemia, which could have been a result of improved bacterial activity in the distal intestine. Moreover, according to studies by Ghaffari et al.^43^, Parveen et al.^44^ and Muthumani and Prabu^45^, silymarin extracted from milk thistle seeds and given orally to rats with liver damage can reduce total plasma bilirubin concentration. However, in all those cases, this effect was accompanied by an improved serum levels of liver enzymes, which was not the case in the present study. Probably, the liver damage was so severe in Zucker rats that it could not be prevented this way.

Our previous study showed that obese Zucker rats are characterized by a severe fatty liver^22,46^ and similar results were obtained in this study. Interestingly, the milk thistle seed supplementation significantly lowered the liver fat by almost 10%; however, the triglyceride and cholesterol contents did not differ among all obese groups (Table 5), which suggests that other liver lipids has to be involved in this reduction, for example phospholipids that are also an important part of the liver^47^. Nevertheless, the lower liver fat deposition could be due to the silymarin content in the seeds. A confirmation of this supposition can be the study by Abdel-Hamid et al.^48^, who reported that oral administration of silymarin to rats with carbon tetrachloride-induced liver fibrosis can reduce fat cell aggregation underneath hepatic parenchyma. Interestingly, despite the reduction in fat content, the liver mass in the O + MTS group was still comparable to that of the other obese groups. According to Sonnenbichler et al.^49^, silymarin stimulates the ribosomal RNA transcription rate and protein synthesis, which can increase the regenerative capacity of the liver. In the present study, obese Zucker rats were also characterized by increased plasma cholesterol, including the HDL and non-HDL fraction (Table 5). Similar disorders in the plasma cholesterol concentrations in obese Zucker rats was observed by Liao et al.^50^. Moreover, de Artinano and Castro^20^ reported that one of the first abnormalities that can be observed in obese Zucker rats is increased plasma lipid and lipoprotein concentrations. Both dietary milk thistle seeds and milk thistle seed oil were able to decrease plasma cholesterol levels (total and its HDL fraction), and their efficiency was similar in this case, which indicates that milk thistle seed oil, and PUFAs in particular were responsible for this ambiguous effect for health. PUFAs are known to stimulate *β*-oxidation of fatty acids in the organism and thus their contribution in the lipoprotein formation is less important^51,52^. Nevertheless, the recent study conducted in our laboratory comparing the effect of poppy seed oil in lean and obese Zucker rats, which has similar fatty acid profile to that of milk thistle seed oil, clearly showed that the reduction of HDL cholesterol under the influence of the oil tested was found only in obese Zucker rats (*fa/fa*) not in lean rats^46^. This indicate that the negative effect of oils on the HDL-cholesterol level is strain-related and specific for obese Zucker rats. Nevertheless, our results are in accordance with the study by Zaki et al.^53^ who observed that a dietary supplementation with only 1% milk thistle seeds can reduce plasma cholesterol concentration in rats with carbon tetrachloride-induced hepatotoxicity, whereas 3% supplementation can normalize the cholesterol to a level found in healthy rats. Our study also shows that the cholesterol-lowering effect of milk thistle seeds is associated with their lipid fraction of which PUFAs are its most important part. However, some authors indicated on a role of silymarin in decreasing plasma cholesterol concentration^54,55^. According to Sobolova et al.^56^, silymarin inhibited cholesterol absorption in rats fed on high cholesterol diet, which resulted in a lower plasma cholesterol concentration. In the present study, the non-HDL cholesterol reduction was significant only in the O + MTS group, which suggests that other components of the seeds, such as silymarin, might also played some modest role in these effects. Moreover, contrary to our previous comparative experiment with the use of hemp seeds and hemp seed oil^23^, milk thistle seeds and milk thistle seed oil did not affect the lipid metabolism on a molecular level and the hepatic mRNA expression of PPAR*α*, PPAR*γ* and SREBP1c was comparable in all obese groups.

## Conclusions

As a result of leptin resistance, obese Zucker rats exhibit a number of metabolic disorders similar to those seen in obese subjects, namely, hyperphagia, hypercholesterolemia, fatty liver and disorders within the distal intestine. This study shows that dietary supplementation with milk thistle seeds and, to a lesser extent, with milk thistle seed oil is able to attenuate some metabolic disorders specific to obese Zucker rats; however, it is not able to affect the obesity development itself. Dietary milk thistle seeds can improve fermentation processes in the distal intestine and liver fat deposition and function, including preventing the development of hyperbilirubinemia, whereas both the seeds and oil have lipid-lowering effects in the blood. This suggest that PUFAs and other oil components of milk thistle seeds are only partly responsible for benefits resulting from their regular consumption. Our findings also indicate that dietary supplementation with seeds rich in nutrients and containing PUFAs and other bioactive compounds can be a useful way to reduce some metabolic disorders associated with genetic obesity and to improve fermentation events in the distal intestine.

## Data Availability

All data generated or analysed during this study are included in this published article.

## Abbreviations

ALP: Alkaline phosphatase
ALT: Alanine transaminase
BW: Body weight
DL: Detection limit
DM: Dry matter
LC: Lean control
MUFAs: Monounsaturated fatty acids
NS: Nonsignificant
OC: Obese control
O + MTO: Obese fed a diet containing milk thistle seed oil
O + MTS: Obese fed a diet containing milk thistle seeds
PPARα: Peroxisome proliferator-activated receptor α
PPARγ: Peroxisome proliferator-activated receptor γ
PSCFA: Short-chain fatty acids of putrefactive origin
PUFAs: Polyunsaturated fatty acids
SCFAs: Short-chain fatty acids
SD: Standard deviation
SEM: Standard error of the mean
SFAs: Saturated fatty acids
SREBP1c: Sterol regulatory element-binding protein 1c
